# Personal Opinions Seem to be the Major Contributor to the Influenza Vaccination Disparity in Sneedville, TN

**DOI:** 10.7759/cureus.7015

**Published:** 2020-02-16

**Authors:** Jacek Bednarz, Daniel Mok, Jan Zieren, Theresa Ferguson, Jose Gomez-Garcia, Jordan Glass, Melanie McCown, Kelcie Smith, Brian Yonish, Aveesha C Bodoe

**Affiliations:** 1 Family Medicine, Lincoln Memorial University - DeBusk College of Osteopathic Medicine, Harrogate, USA; 2 Osteopathic Medicine, Lincoln Memorial University - DeBusk College of Osteopathic Medicine, Harrogate, USA; 3 Wellness Services, Healthy Campus Team, Mount Royal University, Calgary, CAN

**Keywords:** vaccination, vaccine, rural, hesitancy, influenza, barrier, flu

## Abstract

Relevance

Although the seasonal flu vaccine remains the most effective way to prevent the spread of influenza and reduce its associated mortalities, the proportion of individuals receiving the vaccine continues to be an issue in various communities across the United States. The attitudes of residents who live in Sneedville, a small town in a rural northeastern Tennessee, were surveyed.

Objective(s)

To determine the barriers to influenza vaccination in Sneedville, Tennessee and contribute to the literature on why some rural communities across the United States show low influenza vaccination rates.

Materials and Methods

Door-to-door convenience sampling was conducted in Sneedville, TN. Participants were asked to complete a survey consisting of ­­three yes or no demographic questions (one with an option to further elaborate) and nine opinion questions based on a five-point Likert scale (1 = strongly disagree, 2 = disagree, 3 = undecided, 4 = agree, 5 = strongly agree). Participants were not provided any additional details pertaining to the Likert scale questions and were given the option to skip the Likert scale questions. These questions were chosen to gauge the potential structural, socioeconomic, belief, and provider-related barriers to vaccination. Two-tailed independent t-tests were used to compare the Likert scale means for each of the nine opinion questions in those that received the influenza vaccine and those who did not. Univariate analysis was conducted to assess difference in the distribution of Likert responses in vaccinators compared to non-vaccinators.

Results

This project surveyed 172 residents of which 60.5% (104/172) indicated that they did not receive the influenza vaccine for the 2017-2018 flu season. Compared to individuals who vaccinate against the flu, individuals who do not vaccinate against the flu believe the flu shot is not worthwhile and believe the flu shot has a greater chance to make them sick.

Conclusions

This study finds that structural, socioeconomic, and provider-related barriers are not the underlying cause of the low influenza vaccination rates in this rural area. Instead, public opinion on influenza vaccination seems to be the reason for the disparity.

## Introduction

The Centers for Disease Control and Prevention (CDC) estimated that during the 2015-2016 flu season 310,000 Americans were hospitalized for influenza and about 25,000 died from the infection [1]. However, the CDC estimates the deaths maybe two to four times higher when considering individuals who died from influenza, but were not tested, and influenza facilitated deaths resulting from secondary respiratory/cardiovascular complications [2]. Influenza mainly affects the upper respiratory tract and can lead to secondary bacterial infection by *Streptococcus pneumoniae* [3]. The increased mucus production caused by influenza enables the bacteria to proliferate, which further increases morbidity and mortality [4]. The complication may cause death via asphyxiation due to the accumulation of fluid, bacterial debris, liquefied tissue, and neutrophilic infiltrates in the lung parenchyma. Therefore, influenza carries a serious mortal consequence particularly in the immunocompromised. According to the CDC, 5.1 million influenza-related illnesses are thought to have been prevented during 2015-2016 because of the influenza vaccine [2]. As of right now, the influenza vaccine is the most important preventive measure that can be taken to protect oneself from an influenza infection. However, the 2017-2018 influenza season in the United States had the lowest influenza vaccination rate since 2010. During the 2017-2018 influenza season, an estimated 37.1% of adults received the influenza vaccine [5]. In addition, there has been a three-fold increase in the amount of influenza-related hospitalizations and deaths since the 2015-2016 flu season [1]. The most hospitalizations and deaths since 2010 were seen during the 2017-2018 influenza season where 960,000 people were estimated to have been hospitalized and about 79,000 have died [1]. 

Even though the morbidity and mortality of influenza are well known, there has been a recent rise in the rejection of vaccination due to unsubstantiated fears and loss of faith in the utility of vaccination [6-8]. One indication of the skepticism towards vaccination in the United States is the increasing number of parents refusing mandatory vaccinations for their school-aged children. There are 18 states that allow parents to receive a non-medical exemption (NME) for their child if they feel that vaccinations recommended by the* Advisory Committee on Immunization Practices* do not coincide with their beliefs [9]. There has been an increase in NMEs for school children in 12 out of aforementioned 18 states since 2009 [9]. The decline in MMR vaccination in certain parts of the United States, Ireland, France, and the UK has led to outbreaks of measles [9-12]. A recent term in the literature used to describe this phenomenon is *vaccine hesitancy*, which refers to the refusal or delay in acceptance towards vaccination [13]. Vaccine hesitancy is of especial concern in modern times as migration across and within continents has never been so easily accomplished before the appearance of the airplane and automobile. Vaccine hesitancy has become such a large concern that the World Health Organization (WHO) has declared it to be one of the top ten threats to global health in 2019 [14]. A more mobile population facilitates the spread of respiratory viruses like influenza, and as the world’s transportation infrastructure becomes more interconnected, the spread of these viruses such as influenza across the globe will only become easier. As a result, more people are dying from diseases that could have easily been prevented with vaccination.

It is important then to understand the reasons why certain individuals choose not to receive the influenza vaccine so measures can be implemented to encourage vaccination within a community. Past literature has identified categorical barriers to immunization which include structural, socioeconomic/financial, belief/attitude, and provider-related barriers [13,15-21]. Structural barriers are defined as problems in the healthcare infrastructure that hinder an individual’s ability to get vaccinated. This includes inconvenient clinic locations, limited hours of operation, and long wait times to obtain care. Socioeconomic/financial barriers may also play a role depending on an individual’s financial status and/or insurance coverage for treatment. A patient’s beliefs and attitudes towards vaccinations include the thought that the vaccine itself may lead to influenza infection, a disease, or that the vaccine has no benefit. Beliefs and attitudes have been found to be significant barriers to healthcare, especially in the United States [13,17-18,21]. In addition, forgetting to receive an influenza vaccine, or not considering it, have been reported as reasons for not getting vaccinated [21-22]. The presence of these barriers indicates that more work needs to be done to address vaccine hesitancy. Provider related barriers encompass factors such as the quality of the physician-patient relationship, whether or not the provider keeps up-to-date with the patient’s immunization record, and whether or not the physician educates their patients on the health benefits of getting vaccinated. Understanding the major categorical barriers within a community is crucial when determining a plan to increase its vaccination rate.

Rural communities seem to be a population at risk for low influenza vaccination rates due to the aforementioned barriers, thus they pose as epidemic risks in their respective areas [23-24]. The CDC is particularly concerned that rural American adolescents are a population that are getting fewer vaccinations compared to urban counterparts [25]. This study was performed to uncover possible barriers towards vaccination in the small rural town of Sneedville, TN.

## Materials and methods

This study conducted an anonymous door-to-door convenience sampling of Sneedville residents. Informed consent was verbally obtained after a pair of researchers approached a household and introduced themselves as medical students. All Sneedville residents over the age of 18 years willing to participate were eligible for this study. To ensure anonymity, identifiers such as name, date of birth, and address were not collected. Participants were not compensated or rewarded for their participation. The institutional review board of Lincoln Memorial University in Harrogate, Tennessee approved this study (IRB # 709 V.1) on April 27, 2018.

Surveying was completed by eight medical students from Lincoln Memorial University using a paper survey. To be included in the project, participants were asked to respond yes or no to the following two questions and prompt:

 1. Do you have a primary care provider?

2. Did you get the flu shot this year?

3. An authority figure prevented me from getting the flu shot.

If participants answered *yes* to *An authority figure prevented me from getting the flu shot,* space was given to identify the authority figure. If a participant answered *yes* to *An authority figure prevented me from getting the flu shot*, their survey was disqualified in data analysis. This was done because these influential individuals (such as community leaders, or family members) would be the primary reason for the participant's refusal to vaccinate, instead of the aforementioned barriers to vaccination. In addition, surveys that had one or more questions omitted were also removed from data analysis. Nine Likert scale opinion questions were asked, and responses were graded as strongly disagree, somewhat disagree, undecided, somewhat agree, and strongly agree. Participants were given the option to skip any of the questions without giving a reason. Participants were not provided any additional details pertaining to the Likert scale questions. The following Likert scale questions were asked:

1. I forget to get the flu shot during the flu season.

2. The flu shot is affordable.

3. Getting immunized against the flu is worthwhile.

4. I do not get sick often.

5. The flu shot will make me sick.

6. There is a convenient location where I can get the flu shot.

7. Getting the flu shot takes a lot of time.

8. There are convenient times when I can get the flu shot.

9. I don’t want to get the flu shot because of the pain.

The nine Likert scale questions were chosen in order to assess structural, socioeconomical/financial, belief/attitude, and provider-related barriers. Likert means were calculated for each question in vaccinators and non-vaccinators. Independent t-tests were run to compare the Likert means in vaccinators and non-vaccinators. Each Likert response was assigned the following values: strongly disagree = 1, disagree = 2, undecided = 3, agree = 4, and strongly agree = 5. In addition, a Chi-square test was used to assess the difference in the distribution of Likert responses in vaccinators compared to non-vaccinators.

## Results

Over a three-day nonconsecutive surveying period during the 2017-2018 influenza season involving eight student surveyors, a total of 453 residential units were surveyed yielding 181 willing participants. Of the residential units surveyed, 49.9% (226/453) did not have a person readily available to be surveyed, 20.7% (94/453) declined to partake in the survey, and 29.8% (135/453) had one or more respondents per residential unit.

Out of the individuals who did not receive the vaccine, four admitted that an authority figure prevented them to get the vaccine. Of the 181 participants, four were disqualified for listing an authority figure, and five surveys had one or more unanswered questions, leaving 172 usable surveys. Two individuals listed their doctor, one listed their spouse, and one declined to list the authority figure preventing them from vaccination.

A total of 172 usable surveys were collected where 60.5% (104/172) indicated that they did not receive the influenza vaccine for the 2017-2018 flu season. A smaller proportion of non-vaccinators (80.77% (84/104)) have a primary care provider compared to vaccinators (92.65% (63/68)) ($\chi$^2^(1, N=172) =4.670, p=0.031). Univariate analysis also showed statistically significant differences in the distribution of Likert responses with respect to forgetting to get the flu shot ($\chi$^2^(4, N=172) =16.798, p=0.002); affordability of the flu shot ($\chi$^2^(4, N=172) =21.069, p<0.001); worthwhileness of immunization ($\chi$^2^(4, N=172) =57.341, p<0.001); the flu shot causing sickness ($\chi$^2^(4, N=172) =57.348, p<0.001); time to get the flu shot ($\chi$^2^(4, N=172) =17.522, p=0.002); convenient times to get the flu shot ($\chi$^2^(4, N=172) =13.018, p=0.011); and not wanting to get the flu shot because of the pain ($\chi$^2^(4, N=172) =15.173, p=0.004) (Table 1).

**Table 1:** Distribution of survey responses in vaccinators and non-vaccinators The responses from a total of 172 responses were tallied. A Pearson Chi-Square was performed for each question.

	Vaccinators		Non-Vaccinators			
	Count	%	Count	%	Test Statistic	Significance
Do you have a primary care provider						
Yes	63	92.65	84	80.77	4.670	0.031
No	5	7.35	20	19.23		
I forget to get the flu shot during the flu season.						
Strongly disagree	55	80.88	57	54.81	16.798	0.002
Disagree	1	1.47	11	10.58		
Undecided	3	4.41	20	19.23		
Agree	5	7.35	6	5.77		
Strongly agree	4	5.88	10	9.62		
The flu shot is affordable.						
Strongly disagree	0	0.00	4	3.85	21.069	<0.001
Disagree	3	4.41	4	3.85		
Undecided	3	4.41	26	25.00		
Agree	8	11.76	20	19.23		
Strongly agree	54	79.41	50	48.08		
Getting immunized against the flu is worthwhile.						
Strongly disagree	3	4.41	24	23.08	57.341	<0.001
Disagree	0	0.00	8	7.69		
Undecided	4	5.88	28	26.92		
Agree	5	7.35	19	18.27		
Strongly agree	56	82.35	25	24.04		
I do not get sick often.						
Strongly disagree	5	7.35	13	12.50	2.407	0.661
Disagree	8	11.76	14	13.46		
Undecided	1	1.47	4	3.85		
Agree	15	22.06	20	19.23		
Strongly agree	39	57.35	53	50.96		
The flu shot will make me sick.						
Strongly disagree	46	67.65	16	15.38	57.348	<0.001
Disagree	7	10.29	11	10.58		
Undecided	3	4.41	30	28.85		
Agree	8	11.76	13	12.50		
Strongly agree	4	5.88	34	32.69		
There is a convenient location where I can get the flu shot.						
Strongly disagree	1	1.47	5	4.81	7.162	0.067
Disagree	0	0.00	0	0.00		
Undecided	1	1.47	9	8.65		
Agree	4	5.88	11	10.58		
Strongly agree	62	91.18	79	75.96		
Getting the flu shot takes a lot of time.						
Strongly disagree	62	91.18	67	64.42	17.522	0.002
Disagree	2	2.94	21	20.19		
Undecided	2	2.94	13	12.50		
Agree	1	1.47	1	0.96		
Strongly agree	1	1.47	2	1.92		
There are convenient times when I can get the flu shot.						
Strongly disagree	4	5.88	6	5.77	13.018	0.011
Disagree	1	1.47	4	3.85		
Undecided	2	2.94	18	17.31		
Agree	7	10.29	18	17.31		
Strongly agree	54	79.41	58	55.77		
I don't want to get the flu shot because of the pain.						
Strongly disagree	63	92.65	75	72.12	15.173	0.004
Disagree	0	0.00	18	17.31		
Undecided	2	2.94	2	1.92		
Agree	1	1.47	3	2.88		
Strongly agree	2	2.94	6	5.77		

Likert means between vaccinators and non-vaccinators were statistically different for all the questions except, *I do not get sick often* (Table 2). The question, *I forget to get the flu shot during the flu season* scored low Likert means for both vaccinators (M=1.56, SD=1.27) and non-vaccinators (M=2.05, SD=1.36), however statistically lower in vaccinators, t(153) = 2.449, p=0.015. *Getting the flu shot takes a lot of time *scored low among vaccinators (M=1.19, SD=0.70) and non-vaccinators (M=1.56, SD=0.89), however statistically lower in vaccinators, t(164) = 3.017, p=0.003.* I don’t want to get the flu shot because of the pain* scored low among vaccinators (M=1.22, SD=0.83) and non-vaccinators (M=1.53, SD=1.08), however, statistically lower in vaccinators, t(153) = 2.116, p=0.036. The Likert means for vaccinators and non-vaccinators scored high for the questions *The flu shot is affordable* (M=4.66, SD=0.77; M=4.04, SD=1.11; t(170) = 4.349, p<0.001), *There is a convenient location where I can get the flu shot* (M=4.85, SD=0.85; M=4.53, SD=1.06; t(168) = 2.678, p=0.008), and *There are convenient times when I can get the flu shot *(M=4.56, SD=1.056; M=4.13, SD=1.18; t(154) = 2.455, p=0.015), however, statistically higher in vaccinators, respectively (Table 2).

**Table 2:** Likert means of opinion questions answered by vaccinators and non-vaccinators A total of 172 surveys were included in analysis. Independent t-tests were conducted to compare the statistical difference in Likert means between vaccinators and non-vaccinators. Difference in means was found by subtracting the Likert mean of non-vaccinators from the Likert mean of vaccinators.

Question	Likert Mean	Difference in Means	Test Statistic	Significance
I forget to get the flu shot during the flu season.				
Vaccinated	1.56	-0.49	2.449	0.015
Not Vaccinated	2.05			
The flu shot is affordable.				
Vaccinated	4.66	0.62	4.349	<0.001
Not Vaccinated	4.04			
Getting immunized against the flu is worthwhile.				
Vaccinated	4.63	1.5	8.2	<0.001
Not Vaccinated	3.13			
I do not get sick often.				
Vaccinated	4.1	0.27	1.277	0.203
Not Vaccinated	3.83			
The flu shot will make me sick.				
Vaccinated	1.78	-1.59	7.369	<0.001
Not Vaccinated	3.37			
There is a convenient location where I can get the flu shot.				
Vaccinated	4.85	0.32	2.678	0.008
Not Vaccinated	4.53			
Getting the flu shot takes a lot of time.				
Vaccinated	1.19	-0.37	3.017	0.003
Not Vaccinated	1.56			
There are convenient times when I can get the flu shot.				
Vaccinated	4.56	0.43	2.455	0.015
Not Vaccinated	4.13			
I don't want to get the flu shot because of the pain.				
Vaccinated	1.22	-0.31	2.116	0.036
Not Vaccinated	1.53			

There were two questions that had a large dichotomy in opinion among vaccinators and non-vaccinators where the difference in Likert means was greater than 1.0. Vaccinators agreed (M=4.63, SD=0.94) that *Getting immunized against the flu is worthwhile* while non-vaccinators were undecided (M=3.13, SD=1.47), t(170) = 8.200, p<0.001. Vaccinators also strongly disagreed that *The flu shot will make me sick* (M=1.78, SD=1.30) while non-vaccinators were undecided (M=3.37, SD=1.43), t(152) = 7.512, p<0.001 (Table 2).

## Discussion

This project sought to understand what types of barriers Sneedville residents face towards influenza immunization by door-to-door sampling in and around the city limits of Sneedville, Tennessee. A large proportion of the sample chose to not receive the influenza vaccination despite 81.0% (85/105) of non-vaccinators having a primary care provider. *The flu shot makes me sick* and* Getting immunized against the flu is worthwhile *were the two most polarizing opinions based on Likert scores among vaccinators and non-vaccinators. Both groups agreed that the flu shot is affordable, the flu shot is convenient to receive, the flu shot does not take a lot of time to receive, and pain is not a factor for refusing the flu shot. Non-vaccinators disagree that forgetfulness is a major factor for not receiving the flu shot, contrary to what studies examining different populations found [21-22]. This is further supported by both vaccinators and non-vaccinators having similar rates of agreement to the statement,* I forget to get the flu shot during the flu season*. Also, four people out of the 181 surveyed agreed that an authority figure prevented them from receiving the flu shot. Therefore, perception may have more of an influence on whether residents choose to vaccinate instead of financial, structural, or provider-related barriers.

To boost vaccination rates in this community, education towards vaccination needs to be increased. A fraction of non-vaccinators may be swayed to vaccinate if appropriate education and awareness campaigns are implemented in the region. We hypothesize that the best way to sway non-vaccinators is through educational campaigns targeted at those who are undecided. This would be accomplished by addressing specific concerns with respect to the vaccine’s worthwhileness and fear of causing sickness. In addition, almost half of non-vaccinators, 42.3% (44/104), agree and strongly agree that *Getting immunized against the flu is worthwhile* yet choose not to vaccinate (Table 1). These results are encouraging because it seems that residents of the county do not vehemently oppose influenza vaccination; instead they seem to be conflicted for unknown reasons outside the scope of this study. One possible explanation for the county’s low vaccination rate could be that a large proportion of non-vaccinators 44.8% (47/104) agree and strongly agree that *The flu shot will make me sick* (Table 1). However, the rationale for why they believe it will make them sick is not known. Nevertheless, additional education and awareness campaigns could be implemented in this county based on future studies that identify specific reasons why residents have a negative opinion towards influenza vaccination.

The belief that vaccinations are beneficial is a predictor of influenza vaccination in adults in rural communities [15]. This study echoes previous literature, which emphasizes the need for healthcare professionals to focus their attention on educating individuals on the importance of vaccination versus minimizing other barriers such as cost, access to care, or one of the many other barriers. However, past research has suggested that addressing vaccine hesitancy within a community should be tailored to specific concerns and issues; otherwise, the initiative will be ineffective [13].

The limitations of surveying only the Sneedville area in this rural community must be taken into consideration. Residents in isolated areas outside the city limits of Sneedville were not surveyed and could face access issues towards receiving the influenza vaccine that were not represented in the sample. Future studies examining county population distribution with respect to the proportion of individuals who vaccinate may reveal additional barriers this study did not. Additionally, surveying occurred during traditional work hours, which may have been a barrier to accessing more individuals from a potentially key demographic. Future studies that choose to survey at multiple time points during the day, especially those times during which people are traditionally less busy, may reveal additional barriers that this study did not.

Immediate implications of this study extend to primary care providers and other organizations that are stakeholders in vaccination provision, particularly in rural areas. Items in the survey reveal that education and perception are important influencing factors in vaccination acceptance in Sneedville, TN. As such, the community and possibly neighboring areas would benefit from tailored health promotion programming, at the clinical and community level.

## Conclusions

Out of the nine questions addressing different types of barriers to immunization, it was found that the two most polarizing opinions among those who vaccinate and those that do not vaccinate were regarding potential benefits and perceived harm of influenza vaccination. This study also shows that most Sneedville residents have access to care, view the flu shot as affordable, and have a primary care provider. Based on the study, the disparity in influenza vaccination in Sneedville does not seem to be due to structural, socioeconomical, or healthcare provider-related barriers, but rather due to the beliefs about influenza vaccination by the residents. It is our conclusion that educational initiatives specific to concerns with respect to the vaccine’s worthwhileness and fear to cause sickness to be implemented to bolster vaccination rates in the community.

## Appendices

**Table 3:** Raw data For the columns FLUSHOT and PCP *1 *signifies *yes*, and *0* signifies *no*. For the rest of the columns, the numbers correspond to the Likert scale defined in the methods. FLUSHOT = Did you get the flu shot this year?;PCP= Do you have a primary care provider?; FORGET=I forget to get the flu shot during the flu season; AFFORD=The flu shot is affordable; WORTH=Getting immunized against the flu is worthwhile; SCKOFTEN=I do not get sick often; MKMESIC=The flu shot will make me sick; LOCAT=There is a convenient location where I can get the flu shot; LOTTIME= Getting the flu shot takes a lot of time; CONVTIM=There are convenient times when I can get the flu shot; PAIN=I don't want to get the flu shot because of the pain.

FLUSHOT	PCP	FORGET	AFFORD	WORTH	SCKOFTEN	MKMESIC	LOCAT	LOTTIME	CONVTIM	PAIN
1	1	1	5	5	1	1	5	1	4	1
1	1	1	5	5	1	2	5	1	5	1
1	1	1	5	4	5	1	5	1	5	1
1	1	1	5	5	5	1	5	1	4	1
1	1	1	5	5	5	2	5	1	5	1
1	1	4	5	5	5	1	5	1	5	1
1	1	1	5	5	4	1	5	1	5	1
1	1	1	2	5	5	1	5	1	5	1
1	1	1	5	5	4	1	5	1	5	1
1	1	1	5	5	5	1	5	1	5	1
0	1	1	5	2	5	1	5	1	5	5
0	0	1	3	1	5	4	5	2	4	1
0	1	1	5	1	4	5	5	1	5	1
0	1	1	5	4	5	3	5	1	5	1
0	1	1	3	1	5	5	5	4	3	5
0	1	1	4	3	2	3	5	1	5	4
0	1	5	5	3	5	1	5	1	5	1
0	0	3	3	5	5	3	3	3	4	3
1	1	1	5	5	2	2	5	1	5	1
1	1	1	5	5	1	1	5	1	5	1
1	1	1	4	5	4	1	5	1	5	1
1	1	4	5	5	5	1	5	1	5	1
1	1	1	4	3	4	3	5	2	4	1
1	1	1	4	5	5	1	5	1	5	1
1	1	1	5	5	5	1	5	1	5	4
1	1	1	5	5	1	1	5	1	1	1
1	1	1	5	5	5	1	5	1	5	1
1	1	1	5	5	5	1	5	1	5	1
1	1	1	4	5	4	4	5	1	5	1
0	1	3	5	5	5	4	5	1	5	1
0	0	1	4	5	5	1	5	1	5	1
0	1	1	5	5	1	1	5	1	5	1
0	1	1	3	3	5	4	5	1	5	1
0	1	5	5	4	5	3	5	1	5	1
0	1	2	5	3	2	2	5	2	5	1
0	1	4	4	2	5	4	4	2	4	1
0	1	3	4	3	1	2	5	1	5	1
0	1	1	4	2	4	3	4	2	4	1
0	1	4	4	4	4	2	4	2	4	2
0	1	1	5	1	1	3	5	3	5	1
0	1	1	4	1	4	5	4	2	4	2
0	1	1	5	3	5	3	5	2	5	1
0	1	1	5	1	5	4	5	2	3	1
0	1	5	5	5	5	5	5	1	5	1
1	1	1	5	5	5	5	5	5	5	1
1	1	1	5	4	5	1	4	1	4	1
1	1	1	5	5	5	2	5	1	2	1
1	1	5	5	5	4	1	5	1	5	1
1	1	1	5	5	2	1	5	1	5	1
0	0	1	3	5	5	1	5	1	5	1
0	1	5	5	4	2	4	5	1	5	1
0	0	3	5	3	5	5	3	3	3	5
0	0	3	5	3	5	5	3	3	3	5
0	1	1	5	3	5	3	5	1	5	1
0	1	4	3	4	4	2	5	3	3	2
0	1	1	5	2	1	5	5	1	5	1
0	1	1	5	1	1	5	5	1	5	1
0	1	2	2	4	2	2	5	2	5	2
1	1	1	5	5	5	1	5	1	5	1
1	1	1	5	5	5	1	5	1	5	1
1	1	1	3	5	5	5	5	1	5	1
1	1	1	5	5	4	4	5	1	3	1
0	1	1	3	4	4	3	4	3	2	2
0	1	1	4	4	2	5	5	1	3	1
0	1	1	3	1	1	5	5	1	5	1
0	1	1	1	3	4	3	1	1	5	2
0	1	2	1	5	4	1	5	1	1	1
0	1	1	4	4	4	5	5	1	5	1
0	1	1	5	5	1	1	5	1	5	1
0	1	3	5	5	2	3	5	2	5	2
0	1	2	2	1	5	5	5	2	2	2
0	1	1	2	1	2	5	5	1	5	1
0	1	3	3	3	3	3	5	1	5	2
0	0	1	3	1	5	5	4	1	3	4
0	1	1	5	3	1	2	5	1	4	1
0	1	1	3	1	5	3	5	1	1	1
0	1	1	3	4	5	3	5	1	4	5
0	1	3	5	5	5	3	5	2	5	1
0	1	2	3	4	4	3	5	1	4	1
0	1	1	1	5	5	3	5	3	3	1
0	1	1	5	4	5	2	5	1	5	1
0	1	1	4	3	1	3	5	1	5	2
0	1	1	5	1	5	1	5	1	5	1
0	1	1	5	5	5	3	5	1	5	5
0	1	3	3	3	5	4	5	5	5	2
0	1	5	5	5	5	1	5	1	5	1
0	1	5	5	1	5	5	5	1	5	1
0	1	4	5	3	4	3	5	1	5	1
0	1	1	5	3	5	4	5	1	5	1
0	1	1	5	5	1	5	5	1	4	1
0	1	1	5	5	4	1	5	1	5	1
0	1	2	3	3	4	3	4	3	4	1
0	1	3	3	3	4	3	5	3	5	2
0	1	2	2	4	4	1	4	1	1	2
0	1	1	5	4	5	5	5	1	5	1
0	1	1	5	5	2	1	5	1	5	1
0	1	5	5	4	2	2	5	1	5	1
0	1	3	3	3	3	4	3	2	4	2
0	1	2	5	5	3	5	4	2	3	2
0	1	1	5	5	2	4	5	1	1	1
0	1	1	3	1	5	3	5	1	3	1
0	1	1	5	1	5	5	5	1	5	1
0	1	4	4	4	2	5	5	1	4	1
0	1	1	3	1	5	5	5	5	5	1
0	1	5	5	5	1	1	5	1	5	1
0	1	1	5	1	5	5	5	1	5	1
0	1	3	3	4	4	5	3	3	3	1
0	1	5	5	3	5	3	5	3	3	1
0	1	1	5	1	5	5	5	2	5	1
0	1	1	5	1	5	4	5	1	5	1
0	1	3	5	1	2	3	5	2	4	1
0	1	1	4	3	5	5	5	1	3	1
0	1	1	1	5	1	1	5	1	5	1
0	1	3	5	5	5	1	5	1	5	1
0	1	1	3	5	5	1	1	1	1	1
0	1	1	4	1	2	4	5	1	4	1
0	1	3	3	3	5	5	1	1	3	1
0	1	1	5	5	1	5	5	1	5	1
0	0	1	3	3	5	3	5	3	5	1
0	0	1	5	2	5	5	5	1	5	1
0	1	2	5	3	4	5	3	2	2	1
0	1	1	4	2	4	5	5	1	5	1
0	0	5	4	5	3	5	5	1	5	1
0	0	1	4	4	4	2	4	2	2	2
0	0	3	4	2	2	3	4	1	3	2
0	0	2	3	4	5	3	3	2	3	2
0	0	1	3	5	4	3	3	2	4	1
0	0	4	5	3	5	2	5	1	5	1
0	0	1	4	2	5	5	5	1	5	1
0	0	3	3	1	5	5	1	1	1	1
0	0	3	4	3	5	3	1	1	4	4
0	0	2	5	1	5	5	5	1	4	1
0	0	3	5	3	5	4	5	2	3	1
1	0	1	5	5	5	1	5	1	5	1
1	0	2	4	5	5	3	4	2	4	1
1	0	3	3	4	5	1	5	1	1	3
1	0	1	5	5	5	1	5	1	5	1
1	0	4	5	5	5	1	5	1	5	1
1	1	1	2	5	2	4	4	3	4	1
1	1	1	5	5	4	4	5	1	5	1
1	1	3	3	3	3	3	3	3	3	3
1	1	5	5	5	5	1	5	1	1	1
1	1	1	5	5	2	1	5	1	4	1
1	1	1	5	1	4	2	5	1	5	1
1	1	1	5	5	4	2	5	1	5	1
1	1	1	5	5	5	1	5	1	5	1
1	1	4	5	5	2	1	5	1	5	1
1	1	1	5	3	5	1	5	1	5	1
1	1	1	5	1	5	5	5	1	5	1
1	1	1	5	5	5	1	5	1	5	1
1	1	1	5	5	5	1	5	1	5	1
1	1	5	5	5	2	4	5	1	5	1
1	1	5	5	5	5	1	5	1	5	1
1	1	1	5	5	5	1	5	1	5	1
1	1	4	5	3	5	4	5	1	5	1
1	1	1	5	5	2	1	5	1	5	1
1	1	1	5	5	5	1	5	1	5	1
1	1	1	5	5	5	1	5	1	5	1
1	1	1	5	5	5	1	5	1	5	1
1	1	1	5	5	4	1	5	1	5	1
1	1	1	2	5	5	4	5	1	5	1
1	1	1	5	5	4	1	5	1	5	1
1	1	1	5	5	5	1	5	1	5	1
1	1	3	5	5	5	1	5	1	5	1
1	1	1	5	5	4	1	5	1	5	1
1	1	1	5	5	1	1	5	1	5	1
1	1	1	4	5	5	5	5	4	5	5
1	1	1	5	1	4	1	1	1	1	1
1	1	1	4	4	2	1	5	1	5	1
1	1	1	4	4	4	4	4	1	5	1
1	1	1	5	5	5	2	5	1	5	5
0	1	3	4	3	5	2	3	3	3	3
